# Stochastic Modeling of Expression Kinetics Identifies Messenger Half-Lives and Reveals Sequential Waves of Co-ordinated Transcription and Decay

**DOI:** 10.1371/journal.pcbi.1002772

**Published:** 2012-11-08

**Authors:** Filippo Cacace, Paola Paci, Valerio Cusimano, Alfredo Germani, Lorenzo Farina

**Affiliations:** 1 Università Campus Biomedico, Rome, Italy; 2 CNR-Institute of Systems Analysis and Computer Science (IASI), BioMathLab, Rome, Italy; 3 Dipartimento di Ingegneria e Scienze dell'Informazione e Matematica (DISIM), Università de L'Aquila, L'Aquila, Italy; 4 Dipartimento di Informatica e Sistemistica, Sapienza Università di Roma, Rome, Italy; National Center for Biotechnology Information (NCBI), United States of America

## Abstract

The transcriptome in a cell is finely regulated by a large number of molecular mechanisms able to control the balance between mRNA production and degradation. Recent experimental findings have evidenced that fine and specific regulation of degradation is needed for proper orchestration of a global cell response to environmental conditions. We developed a computational technique based on stochastic modeling, to infer condition-specific individual mRNA half-lives directly from gene expression time-courses. Predictions from our method were validated by experimentally measured mRNA decay rates during the intraerythrocytic developmental cycle of *Plasmodium falciparum*. We then applied our methodology to publicly available data on the reproductive and metabolic cycle of budding yeast. Strikingly, our analysis revealed, in all cases, the presence of periodic changes in decay rates of sequentially induced genes and co-ordination strategies between transcription and degradation, thus suggesting a general principle for the proper coordination of transcription and degradation machinery in response to internal and/or external stimuli.

## Author Summary

The amount of a given transcript in a cell is determined by a fine tuned balance of production and degradation in a complex regulatory network. Regulation of transcription controls when transcription occurs and how much mRNA is created, whereas regulation of degradation controls the rate at which messengers are destroyed. The latter mechanism has recently gained attention due to the increasing evidence of its key role in the overall co-ordination of gene expression. A long lifetime of mRNA enables a cell to produce more proteins from that mRNA. By contrast, a short lifetime rapidly alters protein levels in response to changing needs. Measuring mRNA stability is a complex and expensive experiment and, given the condition-specific response of the degradation pathway, it would be desirable to take advantage of the large variety of expression experiments stored in public databases. To this end, we developed a stochastic model to infer each specific mRNA lifetime from gene expression data. Predictions were validated using malaria data. We then applied our methodology to the reproductive and metabolic cycle of budding yeast and found, in all cases, the presence of a general principle for the proper coordination of transcription and degradation machinery.

## Introduction

Appropriate and timely changes in gene expression are essential for cell life. The transcriptome is finely regulated by a large number of molecular mechanisms able to adjust the balance between mRNA production and degradation. Every aspect of transcript life is subject to elaborate control but, traditionally, the focus of the research has been on transcriptional regulation [1]. However, whereas mRNA abundance results from the dynamic interplay between transcription and degradation, the speed by which cells can adjust their mRNA levels is critically dependent on the rate of mRNA turnover [2]. As a result, small changes in mRNA stability may dramatically drive rapid variations of transcript abundance. Efforts to understand the underlying principles of mRNA decay and transcription co-ordination are very important since the balance between transcription and decay influences most, if not all, the cell responses to endogenous and exogenous signals [3].

The current widespread interest in this topic has been fostered by the finding of specific regulatory mechanisms of mRNA stability such as, for example, RNA binding proteins [4], [5] and small RNAs [6]. Regulation of transcript stability cannot be considered a simple “disposal system” but a sophisticated tool for the proper orchestration of the global cell response to internal and external stimuli [6]. Remarkably, a key role of mRNA stability has been reported in cancer, inflammatory diseases and Alzheimer's [7]. In recent years there has been a surge in empirical studies that measured, on a genome-wide scale in a variety of environmental conditions, messenger half-lives of many organisms, including plants [8] mammals [9] and fungi [2]. The discovery of such new regulatory layer has clarified that, in order to obtain a clear picture of the underlying regulatory machinery, it is necessary to complement the traditional time-course experiment measuring the cell transcriptional response under certain conditions far from steady state with decay rates data under the *same* condition [10].

**Figure 1:**
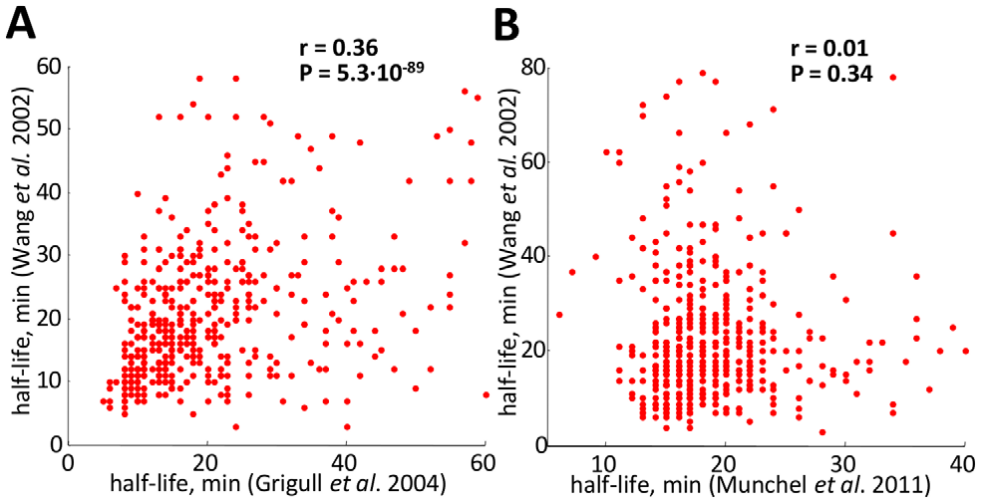
Basic comparison statistics among the yeast *S. cerevisiae* mRNA half-lives during asynchronous growth measured by three independent laboratories. Three genome-wide studies considered are: Grigull *et al.* [12], Wang *et al.* [11] and Munchel *et al.* [2]. (A) Scatterplot of Wang *et al.* and Grigull *et al.* datasets; (B) Scatterplot of Munchel *et al.* and Wang *et al.* datasets.

Experimental procedures for the evaluation of mRNA decay rates are based on measuring gene expression upon inhibition of transcription [11]–[13] or on pulse-chase RNA labeling protocols [2], [13]–[15]. Such protocols are very critical (see Figure 1 for a comparison among different studies summarized in Table 1), since, for instance, transcriptional shut-off blocks growth and has a profound effect on cellular physiology, as well as on mRNA metabolism [2]. In fact, Wang *et al.* [11] and Grigull *et al.* [12] datasets show a low value of the Pearson correlation (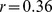), and no correlation at all can be found (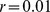) between Munchel *et al.* [2] and Wang *et al.* [11] datasets (see Figure 1A and Figure 1B respectively). Despite the same experimental conditions (asynchronous growth), the two half-life independent measurements obtained by Wang *et al.* and Munchel *et al.* are uncorrelated, probably due to differences in the shut off protocol (pulse chase for [2] and thermal inactivation for [11]), whereas Grigull *et al.* and Wang *et al.* appears significantly correlated, probably due to the same shut off protocol used (thermal inactivation).

**Table 1:** Half-lives experimental measurements.

*Organism*	*Description*	*Publication*
	2774 genes, synchronized by sorbitol treatments,	
Plasmodium IDC	transcriptional shut-off by actinomicin D	Shock et al. (2007)
	5492 genes, asynchronous growth in 2% glucose, pulse-	
Yeast	chase labeling protocol	Munchel et al. (2011)
	4680 genes, asynchronous growth in YPD medium,	
Yeast	transcriptional shut off by thermal inactivation	Wang et al. (2002)
	2867 genes, asynchronous growth in YPD medium,	
Yeast	transcriptional shut off by thermal inactivation	Grigull et al. (2002)

It has been shown that genes having the same biological function [11], [12] are likely to share similar half-life values. Consistently, by averaging using functional groups, we found an increase in correlation between Wang *et al.* and Grigull *et al.* datasets (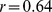, see Supplementary Figure S1A), and still no correlation between Munchel *et al.* and Wang *et al.* datasets (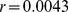, see Supplementary Figure S1B).

Here, we developed a stochastic computational model of the expression kinetics to identify condition-specific mRNA stabilities which makes use only of experimental mRNA time profiles. We also assumed that degradation rates are gene-specific but approximately constant over the experiment time course. Predictions of our algorithm, termed DRAGON (Decay RAtes from Gene expressiON), were validated on experimental mRNA abundance [16] and turnover [17] data, both collected during the Intraerythrocytic Developmental Cycle (IDC) of *Plasmodium falciparum*. The estimations were in line with the experimental measurements. Remarkably, the DRAGON estimated half-lives were consistent with the finding of a peculiar pattern of mean half-life values along the wave of sequentially induced genes in subsequent stages of *P. falciparum* development. We also applied our methodology to public time-series datasets for which half-lives data, under the same experimental conditions, have not been experimentally measured. In particular we focused on budding yeast reproductive [18], [19] and metabolic cycle data [20]. In fact, for the yeast *Saccharomyces cerevisiae*, only half-life data under asynchronous growth are publicly available [2], [11], [12]. Our study showed the presence of the same periodic pattern of mean half-life values in all datasets, thus suggesting that such behavior may be a general feature, not limited to the *Plasmodium falciparum* IDC.

### mRNA kinetics and half-life

**Figure 2:**
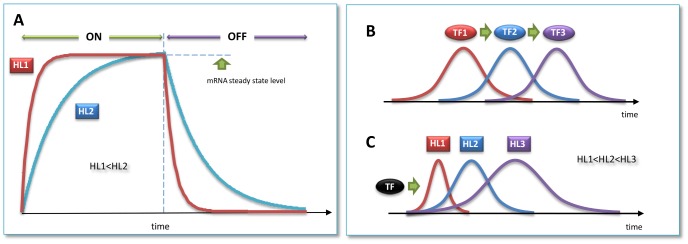
Kinetics of gene induction. Panel A shows *in silico* experiments to illustrate some basic features of gene induction kinetics. The “ON” and “OFF” regions correspond to the turning “ON” or “OFF” of the promoter activity. (A) Induction kinetic of transcripts having the same steady-state concentration, but different half-lives and synthesis rates reaching the same steady state value. The time profile plotted in red corresponds to an unstable transcript and displays a fast induction and relaxation profile. By contrast, the blue one has an higher half-life value, resulting in a slower response. (B) Cascade of transcription factors resulting in waves of sequentially induced genes. The timing of expression peaks is modulated by transcriptional serial regulation. (C) Sequentially induced genes generated by a single transcription factor and a stability “gradient”. The timing of expression peaks is modulated by post-transcriptional regulation. Early induced genes are those with a low half-life value, late induced ones are those with an high half-life value.

Experimental evidence suggest that the majority of mRNAs are degraded with a first-order decay rate [2], [21]. This allows to characterize mRNA disappearance time profiles by a first-order rate equation
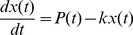
(1)where 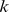 is the decay rate (or half-life 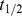, with 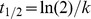), 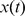 is the mRNA concentration and 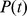 is the promoter activity (the rate of production of new mRNAs). It is worth noting that, the degradation rate 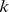 cannot be estimated from the concentration time profile 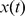 for a single gene, since the term 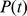 is not usually available in the typical time-course microarray experiment. The measurement of the promoter activity time profile would require additional experiments (such as those described in [15]) but, in this paper, we will assume that only mRNA abundance time-series data are available. At steady-state 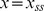 and 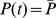 are constant so that 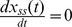 and, consequently, we obtain
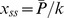
(2)From the above equation, it is clear that at steady-state an increase (decrease) in mRNA concentration can be produced either by an increase (decrease) of transcription or by a decreased (increased) value of the decay rate: the two regulatory strategies have therefore an equivalent outcome. As a result, from steady-states measurements, it is hopeless to reveal the relative contribution of transcription and degradation and, most importantly, their co-ordinated activity as well. By contrast, the whole kinetics of induction and relaxation, as measured by time-courses experiments, depends on the degradation and production rate in different ways: increasing (decreasing) the production rate results in a proportionally increased (decreased) mRNA abundance, whereas the rise time (*i.e.* the time required for the response to rise from 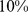 to 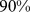 of its final value) is not affected. Increasing the decay rate results in a faster rise time both in the induction and relaxation phases, whereas a decrease results in slower rise time [10]. This key point is illustrated in Figure 2A and in Supplementary Figures S2A and S2B.

Another important consequence of half-life specificity is the regulation of the timing of gene induction, as pointed out by Elkon *et al.* [22]. In fact, an expression wave, *i.e.* the sequential activation of genes, is usually interpreted as resulting from the corresponding activation of a multi-step transcription factors cascade (as illustrated in Figure 2B). Whereas such mechanism is certainly very important, there is also an alternative way to obtain an expression wave by means of a “stability gradient”. As illustrated in Figure 2C, a single transcription factor may initiate transcription of a set of target genes and their peak of induction can be modulated by a stability “gradient”, *i.e.* by specifically adjusted decay rates. More precisely, early induced genes would have short half-lives and late responding genes would have long half-lives. Clearly, both mechanism may well act in cells, thus generating a wide spectrum of responses.

Time-courses are a very common design for microarray analysis, which allows researchers to follow the dynamics of the cellular response to perturbations [22]. Such data are available for a very large number of experimental conditions and organisms: only the Stanford Microarray Database includes to date 1545 time course data sets. Among the examples later illustrated in the paper, it is worth mentioning the genome-wide gene expression time-series obtained during the reproductive cell cycle [18], [19], the metabolic cycle [20] and the *P. falciparum* IDC [16]. The time-series datasets used in this paper are summarized in Table 2.

**Table 2:** Gene expression time-series experimental measurements.

*Experiment*	*Description*	*Publication*
	4488 genes, 1 cycle, 48 time points, 1 sample at 1 hr	
Plasmodium IDC	interval, synchronized by sorbitol treatments	Bozdech et al. (2003)
	4775 genes, 2 cycles, 25 time points, 1 sample at 5 min	
Yeast cell cycle	interval, alpha-factor synchronization	Pramila et al. (2006)
	1271 genes, 2 cycles, 15 time points, 1 sample at 16 min	
Yeast cell cycle	interval, synchronized by centrifugal elutriation	Orlando et al. (2008)
	6441 genes, 3 cycles, 36 time points, 1 sample at 25 min	
Yeast metabolic-cycle	interval, spontaneous synchronization	Tu et al. (2005)

### DRAGON–an algorithm for half-life estimation

The goal of the DRAGON methodology is to derive a robust estimate of each mRNA species half-life starting from all available gene expression pairs. The rationale for the algorithm mainly draws on properties of pairs exhibiting a certain degree of common promoter activity (as in [23]). Besides, DRAGON infers common promoter activity using a statistical model that simulates both gene-specific and common effects.

The rate of change of mRNA concentration for a generic pair of genes, say gene 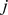 and gene 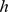, is:
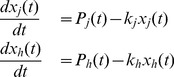
(3)where the symbols 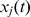 and 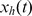 represent the mRNA time profiles of the gene pair 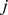 and 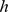, 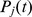 and 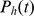 are the promoters activity, and 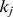 and 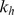 are the degradation rate of mRNA of gene 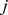 and 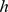, respectively. The terms 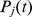, 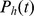 are not known since we considered the case in which only mRNA abundance is measured.

We modeled promoter activities as the sum of two terms, the first one common to the pair and the other one specific for each gene:
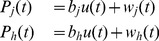
(4)where 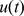 is the common part, scaled by constants 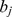 and 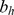, whereas 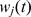 and 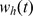 are gene specific independent stochastic processes with zero mean, that is 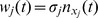, 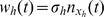. Equations (3)–(4) encompass the case of:

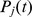
, 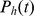 fully correlated (correlation 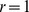) for which 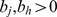 and 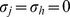

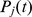
, 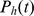 partially correlated (correlation 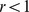) for which 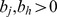 and 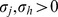

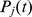
, 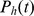 un-correlated (correlation 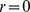) for which either 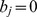 or 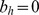.

Equations (3)–(4) can therefore be written for all available gene pairs; thus, for a set of 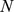 genes, we have 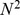 pairs to analyze. For each gene pair DRAGON provides an estimate of the time profile of 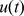, of all the parameters, 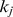, 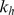, 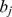, 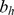, and the covariance matrix of the stochastic processes. For each gene we therefore have 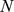 estimates of the decay rate 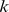, one for each pair containing that gene.

Notice that equations (3)–(4) yield a couple of linear stochastic differential equations. Since measurements of mRNA concentrations are available only at given time points, it is necessary to transform (3)–(4) in a couple of discrete stochastic equations. The exact discretization of (3)–(4) is possible since they are linear [24]. The *Kalman filter* [25] is used on the resulting discrete equations and a maximum likelihood algorithm is exploited to generate the best possible estimate of the parameters.

A complete description of the mathematical model and of the discretization and parameters estimation procedure is given in the paragraph *Stochastic modeling of expression kinetics and Kalman filtering* of the *Materials and Methods* section.

## Results

### Performance evaluation on malaria IDC experimental data

The IDC is characterized by four morphologic stages: ring, trophozoite, schizont and late schizont. The cycle begins with the red cells invasion by merozoites followed by a remodeling of the host cell in the ring stage [16]. The merozoites then develop into trophozoites. During the schizont stage, after a period of growth, the trophozoite undergoes an asexual dividing process and the parasite is ready for the next round of invasion by new merozoites (late schizont phase).

Bozdech *et al.* [16], using microarrays, measured genome-wide mRNA abundance profiles across 48 h during one cycle of *P. falciparum* IDC, collecting one sample per hour. Later on, Shock *et al.* [17] measured mRNA half-lives of 2774 transcripts of the IDC using chemical inhibitors to reach transcriptional shut-off.

**Figure 3:**
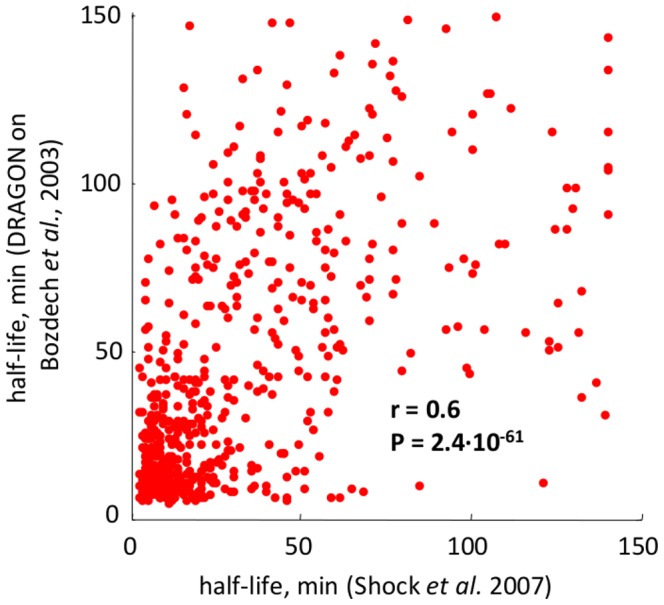
DRAGON algorithm validation using *P. falciparum* IDC data. Scatterplot of mRNA half-lives for 616 genes estimated by DRAGON *versus* experimentally measured by Shock *et al.* [17].

The simultaneous availability of gene expression and decay data during the same biological process (IDC) represents a natural test bed for the validation of the DRAGON algorithm. Therefore, we applied DRAGON on Bozdech *et al.* dataset to obtain mRNA stability estimations (provided in Supplementary Table S6) to be compared with Shock *et al.* measurements for performance evaluation. The resulting Pearson correlation between *in vitro* and *in silico* measures is 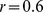 (P value 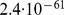), and the first principal component accounts for 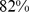 of the variability, thus showing a good performance for DRAGON algorithm (see Figure 3). However, since gene expression and decay data have been measured by different groups, we can speculate that 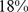 of unexplained variability may be partly due to inherent biological variability and to transcriptional inhibition stress. As further analysis, we computed average mRNA half-lives in both studies for functional categories (see Supplementary Figure S3). We found that the two studies are in better accordance when half-lives are averaged for all genes within any given functional category (Pearson correlation 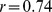).

**Figure 4:**
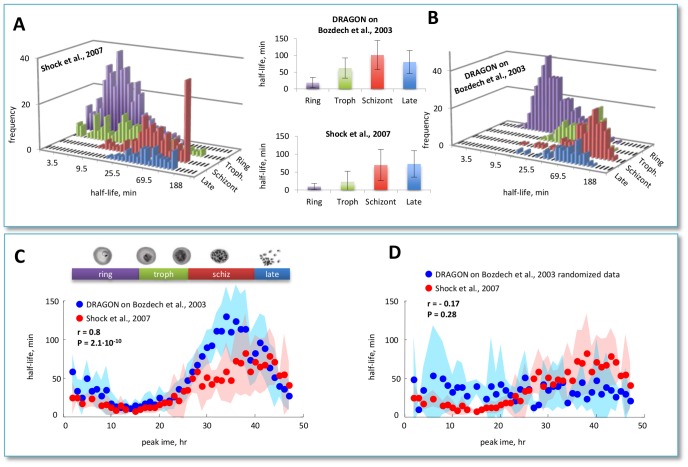
Periodic behavior of average half-lives of sequentially induced genes in *P. falciparum* IDC. (A) Histograms of mRNA half-lives for genes induced at each stage of the *P. falciparum* IDC as experimentally measured by Shock *et al.* and (B) estimated by DRAGON algorithm. The inset panels show mean and standard deviation of half-lives during each stage. Both studies show an increase of average transcript stabilities of sequentially induced genes during *P. falciparum* IDC. (C) Average experimental and estimated half-life values (red and blue dots, respectively) corresponding to genes having the same expression peak timing, indicated on the x-axis. Standard deviations are drawn as pale blue and pale red stripes. The two curves both show a maximum peak of average half-life value during the schizont stage and a minimal value during the ring stage. A sharp increase of average half-life occurs during the trophozoite stage. The Person correlation between experimental and DRAGON estimated curve is 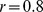, thus showing a good agreement between the two studies. (D) Effect of randomizing the gene expression matrix on DRAGON estimated half-lives.

Remarkably, Shock *et al.* in [17], found progressive stage-dependent average increases in mRNA stability and suggested such phenomenon to be a major determinant of mRNA accumulation (see Figure 4A). The same feature is also found using DRAGON estimated half-lives (see Figure 4B). To investigate in further detail the behavior of average half-life of genes sequentially induced during IDC, we computed for each gene the time point corresponding to its peak of expression (see the *Data processing* paragraph of the *Materials and Methods* section for details) and selected 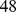 groups of genes having peak of expression at each hourly time points over the 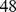 hours monitored by Shock *et al.* For each gene group we computed half-lives mean and standard deviation and found a high correlation with the corresponding curve obtained using experimental data (Pearson correlation 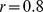, P value 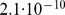; see Figure 4C). Early responding genes are characterized by high instability, whereas late responders are more stable, as also reported by Elkon *et al.* in [22] when studying mammalian cells. A possible explanation for the presence of stable mRNAs at the schizont stage, suggested by Shock *et al.*, is that it may be important for the merozoite to receive a carefully regulated “starting package”, that would allow rapid activation of the IDC following the next round of invasion [17]. By contrast, the initial low mRNA stability values may be an indication of the fast dynamic remodeling after merozoite invasion [17]. To evaluate the probability of obtaining such behavior by chance, we randomized the gene expression matrix and used DRAGON to estimate half-lives (see Figure 4D). Consistently, the estimation of half-lives using random data does not produce any correlation with experimental data (Pearson correlation 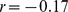).

### Half-lives estimation during reproductive cycle in *S. cerevisiae*

**Figure 5:**
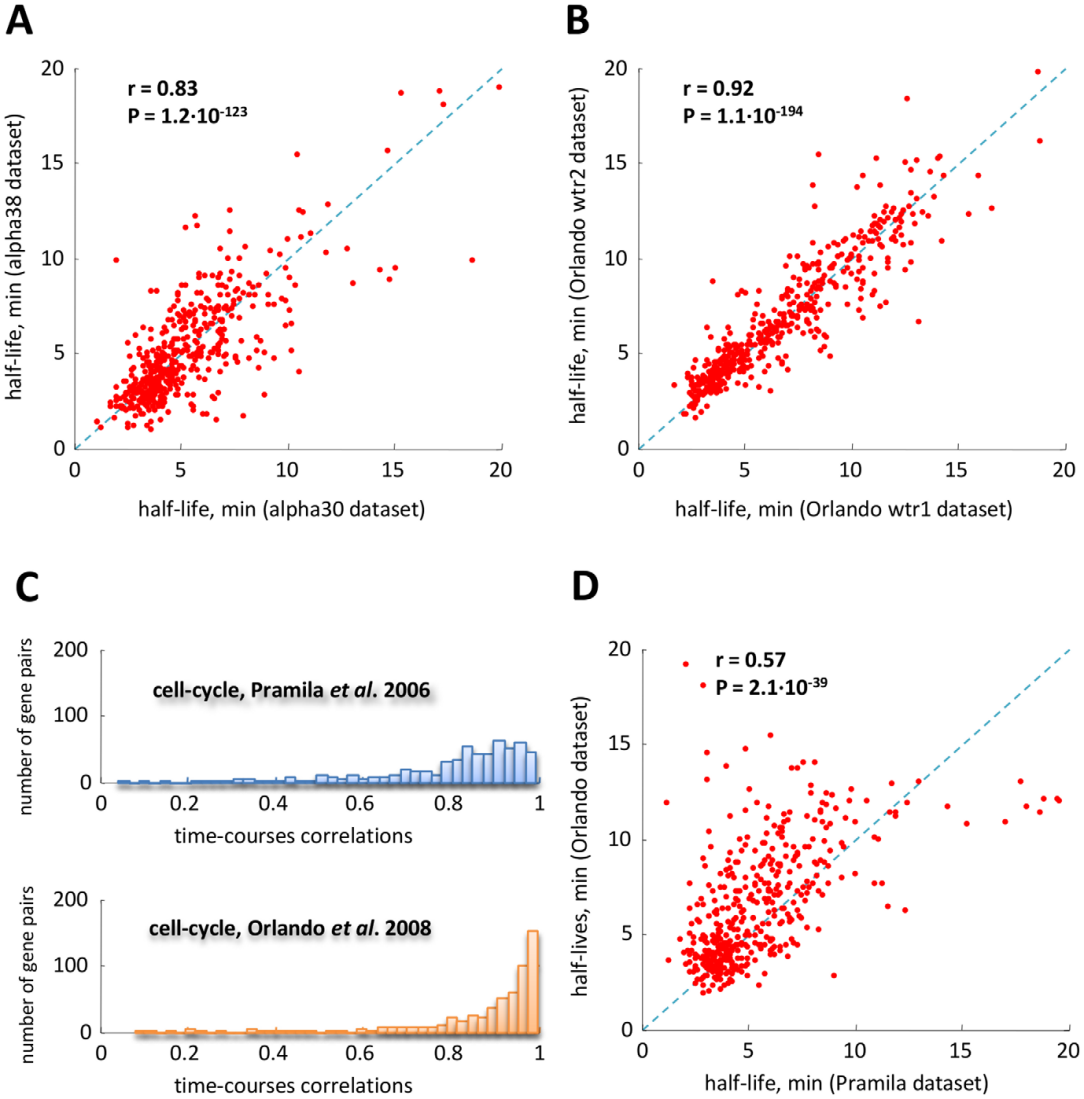
DRAGON performance over 569 cell cycle regulated genes from the Pramila and Orlando datasets. (A) Scatterplot of the DRAGON estimated half-lives using two replicates taken from Pramila *et al.* dataset [18] (denoted by alpha30 and alpha38). (B) Scatterplot of the DRAGON estimated half-lives using two replicates taken from Orlando *et al.* dataset [19] (denoted by Orlando wtr1 and Orlando wtr2). (C) Histograms of the Pearson correlation values between time series relative to each gene in two replicates. Orlando *et al.* dataset shows a better consistency between replicates with respect to the Pramila *et al.* dataset. (D) Scatterplot of the DRAGON estimated half-lives using the Pramila *et al.* [18] and the Orlando *et al.* dataset [19]. The half-lives obtained by replicate datasets have been averaged. The half-lives estimated using Orlando dataset show slightly higher values with respect to those obtained using Pramila dataset, as shown by the deviation from the bisector line (dashed blue line). This is consistent with the slower cell cycle in Orlando experiment (2 hours) compared to that of Pramila experiment (1 hour).

Gene expression during yeast cell cycle has been recently measured by Pramila *et al.* [18] using alpha-factor synchronization and by Orlando *et al.* [19] using centrifugal elutriation for synchronization. We obtained a high consistency of DRAGON estimations using data for 569 transcripts over replicate datasets (Pearson correlation 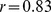 for Pramila *et al.* dataset and Pearson correlation 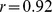 for the Orlando *et al.* dataset; see Figure 5A–B). The larger variability in half-lives estimations may be explained by the inconsistencies between replicate time-series in the Pramila *et al.* dataset with respect to the Orlando *et al.* dataset (see Figure 5C). All half-lives estimations obtained with the DRAGON algorithm are provided in Supplementary Tables S1 and S2 (Pramila) and in Supplementary Tables S3 and S4 (Orlando). Notwithstanding significant differences in synchronization procedures, we also found a high correlation of DRAGON half-lives estimations over the two datasets (Pearson correlation 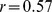, P value 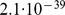; see Figure 5D) where the first principal component accounts for 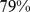 of the overall variability. We can speculate that 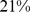 of unexplained variability may be partly due to the different synchronization methods used. In fact, Orlando *et al.* obtained a cell cycle duration of about 2 hours, 8 samples per cycle [19], whereas Pramila *et al.* obtained a cell cycle duration of about 1 hour, 12 samples per cycle. Consistently, most of the transcripts during the slower cycle display higher half-lives when compared to the fastest cycle (see Figure 5D).

### GO annotations of genes with extreme half-lives in *S. cerevisiae*

In this paragraph we briefly discuss functional annotations (done using GOrilla software [26]) of novel predicted half-lives provided by DRAGON algorithm using yeast reproductive and metabolic cycle time series. For the yeast cell cycle we normalized the half-life log-distribution (Z-score), for each dataset, and then computed the geometric mean to obtain a single half-life value for each gene. Notably, the averaging has the effect of reducing the impact of the different synchronization stress response. The list of half-lives normalized values (geometric mean value equal to 1) for common genes to all datasets is provided as Supplementary Table S7 in the *Half-life estimation* paragraph of the *Materials and Methods* section.

Unstable genes are enriched with replication fork complex (p-value 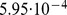) and stable genes (histones HA1-2,HB1-2) are enriched with nucleosome (p-value 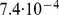). This is consistent with the need of producing a large number of histones during DNA replication process so that stable histone mRNAs contribute to a higher translation efficiency. Moreover, DNA replication timing requires first the formation of the replication fork, then the production of the needed histones for chromatin assembling: such temporal sequence of events is consistent with a rapid turnover of the replication complex genes and a slow turnover of the histone genes (see Supplementary Figure S4). Among unstable genes we also found the G1/S transition cyclins and among stable ones we found G2/M transition cyclins (see Supplementary Figure S5). In this case, the temporal sequence of events is the progression of the cell cycle from DNA replication to mitosis.

For the yeast metabolic cycle (half-lives estimations using DRAGON algorithm are provided in Supplementary Table S5) we found many stable mRNA species involved in the organic acid and arginine metabolism and protein catabolic processes. Among unstable messengers we found genes involved in DNA repair (p-value 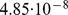), DNA metabolism (p-value 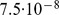) and chromatin silencing (p-value 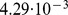).

## Discussion

### Periodic behavior of average half-lives of sequentially induced genes

The increasing pattern of average half-life found during *P. falciparum* IDC (shown in Figure 4A) motivated us to investigate whether a periodicity could be found also in other cyclical biological processes. We focused on the reproductive cell cycle and the metabolic cycle in *Saccharomyces cerevisiae*, for which high resolution time series measurements are available on public repositories (see Table 2).

**Figure 6:**
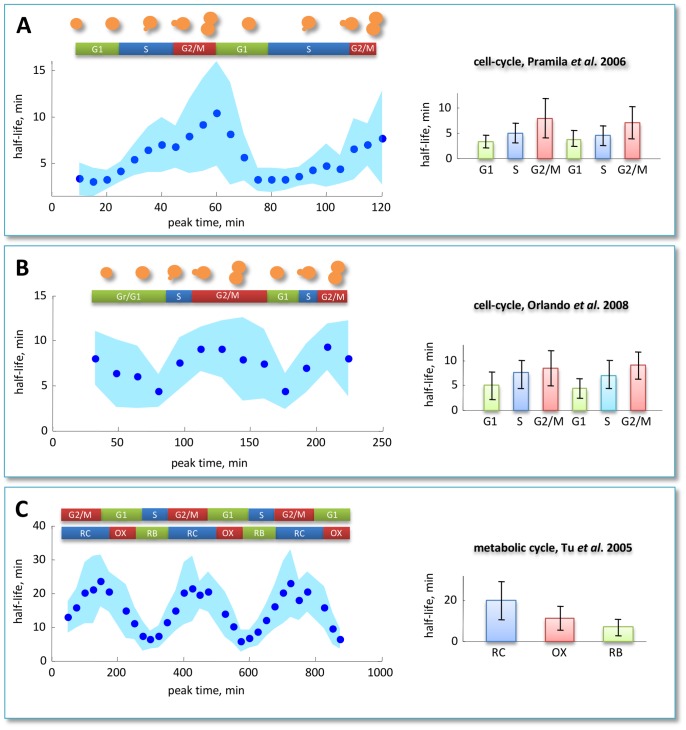
Periodic behavior of average half-life during the reproductive and metabolic cycle. Average DRAGON estimated messenger half-life values corresponding to genes having the same expression peak timing, indicated on the x-axis (dark blue points). Standard deviations are drawn as pale blue stripes. (A) yeast cell cycle using Pramila dataset, (B) yeast cell cycle using Orlando dataset and (C) yeast metabolic cycle. Strikingly, in all datasets the maximal average half-life is attained for genes induced during G2/M phase, including the metabolic cycle. The minimal average half-life, in all datasets, is attained at the G1/S transition phase. The bar charts show mean and standard deviation of half-lives during each stage.

To study if a periodic pattern of average half-life of sequentially induced genes exists along the cell cycle progression, for each gene we computed the time points at which maximal expression is attained (see the *Data processing* paragraph of the *Materials and Methods* section for details). Thus, we obtained, for each time point, the list of genes having expression peak value at that time and computed the corresponding mean and variance of DRAGON estimated half-lives values. Indeed, we found a cyclic behavior along sequentially induced genes in both datasets (see Figure 6A for the Pramila *et al.* dataset and Figure 6B for the Orlando *et al.* dataset). Synchronization methods, cell cycle duration and number of samples are different between the two cited studies, but, reassuringly, the phases of the cell cycle at which mean half-life is minimal or maximal is consistent. In fact, for both datasets we observed a cyclical increase of mean half-life from G1 phase to M phase and a subsequent decrease back to G1. The figure clarifies that the minimal mean half-life is reached at the G1/S transition, whereas the maximal value correspond to the M/G2 phase for both cycles and datasets. The latter is consistent with the observation that, in higher eukaryotes, mitosis is accompanied by global repression of nuclear RNA synthesis [27], indicating that mRNAs must be stable to be inherited from daughter cells.

The yeast metabolic cycle has been recently studied by Tu *et al.* [20] using a continuous culture system, after a brief starvation period, the culture spontaneously began persistent respiratory cycles of about 5 hours. In the same study, a genome-wide microarray gene expression measurement was performed. Samples were taken every 25 minutes for 3 consecutive cycles. Using DRAGON algorithm we estimated half-lives using data of 1043 transcripts. Surprisingly, also in this case we found a cyclical pattern for mean half-life of sequentially induced genes. The maximum peak is located at the RC phase and the minimum peak located at RB phase (see Figure 6C).

### Integrated analysis–sequential waves of co-ordinated transcription and decay

**Figure 7:**
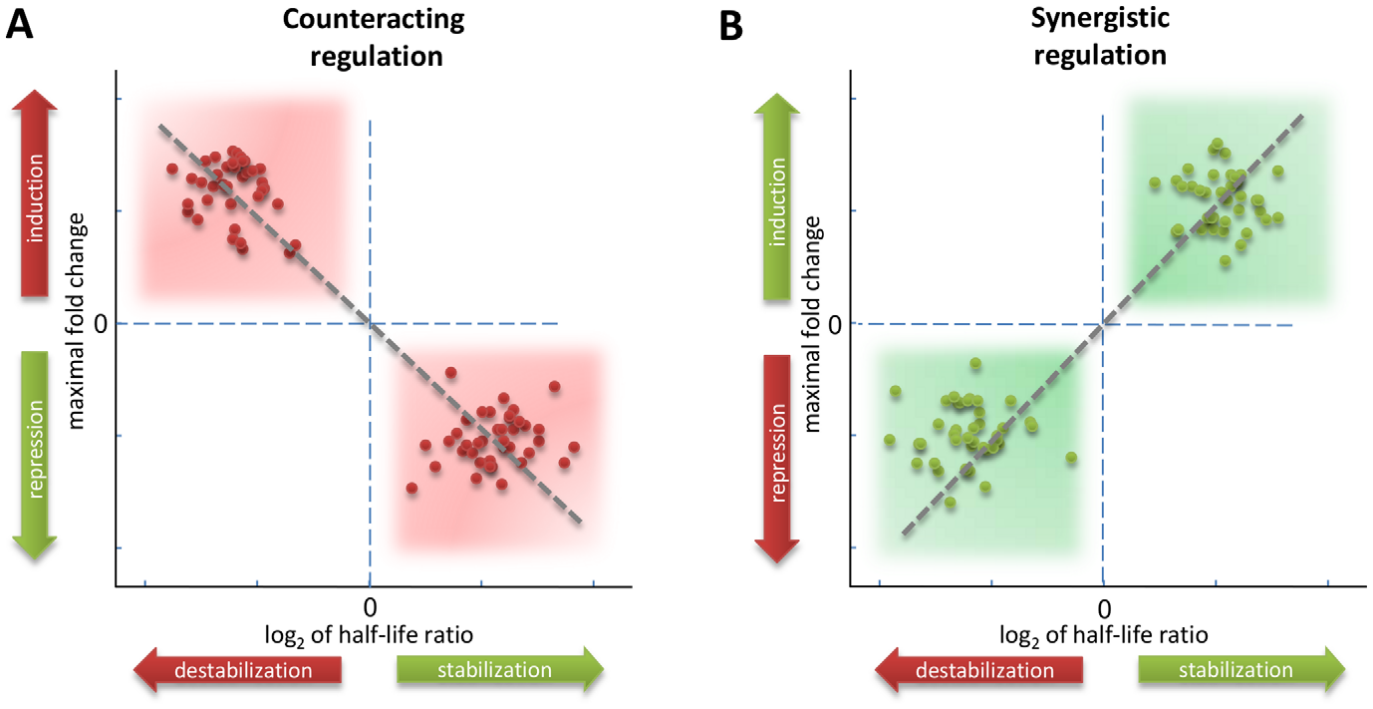
Illustration of the *counteracting* and *synergistic* regulatory strategy. The stability/folding diagrams (SF), introduced by Shalem *et al.* in [10], show the change in mRNA stability relative to the average value plotted against the maximal fold change. (A) Counteracting strategy (negative correlation): induced genes are destabilized and repressed genes are stabilized. (B) Synergistic strategy (positive correlation): induced genes are stabilized and repressed genes are destabilized.

Recently, the appearance of a number of studies has revealed the fundamental role of stability regulation in shaping appropriate cell response [1]. A key point has been recently addressed by Shalem *et al.* [10], who have shown the dynamic co-ordinated interplay between transcription and degradation. They have found in yeast two basic regulatory strategies in response to stress. More precisely, they measured changes of mRNA abundance and decay rates in a yeast population subjected to oxidative and DNA damage stress. By grouping genes according to the time point at which the maximal (minimal) fold change is attained and combining normalized (mean and variance) mRNA abundance and decay rate data, they constructed a “stability *versus* folding” (SF) diagram where change in mRNA stability relative to a reference state (mean value in our case) is plotted against the maximal fold change. Using yeast expression time-course data obtained in response to an oxidative stress and a DNA damage, they were able to reveal two different strategies: a) a “counteracting regulation” strategy (see Figure 7A), characterized by genes in which an increase (decrease) in degradation rates counteracts a increase (decrease) in mRNA abundance, *i.e.* repressed genes are stabilized and induced genes are destabilized; b) a “synergistic regulation” strategy (see Figure 7B), characterized by genes in which an increase (decrease) in degradation rates is associated with an decrease (increase) in mRNA abundance, *i.e.* induced genes are stabilized and repressed genes are destabilized.

Shalem *et al.* also found that, progressing from early time points forward, the negative correlation (counteracting) was replaced with a positive correlation (synergistic). Such co-ordination strategy may permit crosstalk between different steps of mRNA biogenesis, providing a mechanism to control the order and timing of events [28]. The work of Shalem *et al.* has shown the importance of combining expression data with decay rates under the same experimental condition to reveal the underlying strategy of co-ordination of the two “regulatory arms”, namely transcription and degradation. Uncovering such relationships is certainly a fundamental task, since the underlying reciprocal influences between mRNA production and degradation are largely unexplored [10]. The DRAGON algorithm, by estimating half-lives directly from gene expression data under specific conditions, allows the computational integration of mRNA abundance and decay rates data, making this powerful combined analysis possible when experimentally measured half-lives are not available.

**Figure 8:**
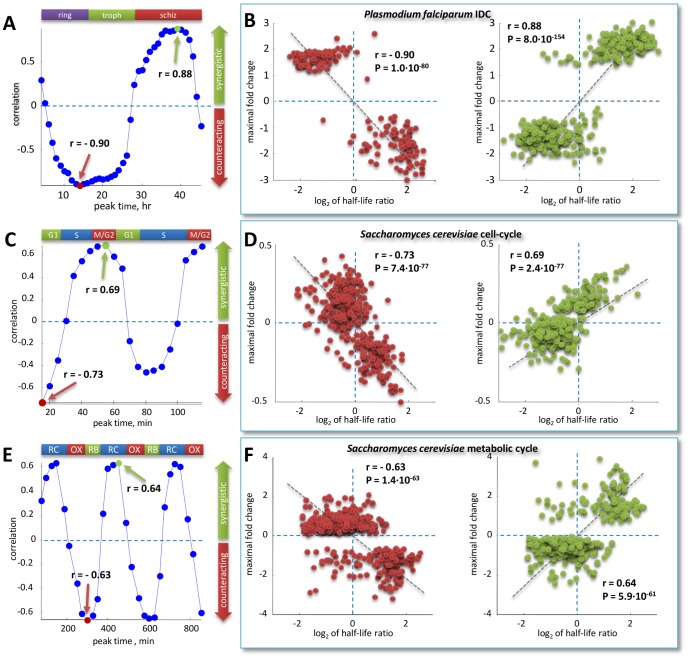
Temporal progress on the stability/folding (SF) diagrams. In all cases under study, we found a progressive shift from an inverse (counteracting) to a direct (synergistic) relationship. (A) *Plasmodium falciparum* IDC, (C) yeast cell cycle (Pramila *et al.* dataset) and (E) yeast metabolic cycle. In panels A,C and E each blue dot corresponds to a Pearson correlation of the SF diagram at the peak time point indicated on the x-axis, for the three datasets. In panels B,D and F the SF diagrams corresponding to the correlation values indicated by arrows in panels A,C and E are displayed. The arrows point to maximal negative (red dots in panels B,D,F and red arrows in panels A,C,E) and maximal positive correlation values (green dots in panels B,D,F and corresponding green arrows in panels A,C,E).

We computed SF diagrams for *P. falciparum* IDC, yeast cell cycle (Pramila *et al.* dataset) and metabolic cycle (shown in Figure 8). In panels A,C and E each blue dot corresponds to a Pearson correlation of the SF diagram at the peak time point indicated on the x-axis, for the three datasets. In panels B,D and F the SF diagrams corresponding to the correlation values indicated by the arrows in panels A,C and E are displayed. The arrows point to maximal negative (red dots in panels B,D,F and red arrows in panels A,C,E) and maximal positive correlation values (green dots in panels B,D,F and green arrows in panels A,C,E).

Strikingly, in all cases we reached the same conclusions of Shalem *et al.*, namely we found that early induced genes show counteracting regulation, whereas late induced genes show a synergistic regulation.

### Advantages and disadvantages of the method

The main advantage of the DRAGON algorithm consists in the estimation of the mRNA half-lives directly from gene expression time-course during condition-specific experiments. Moreover it estimates the correlation among promoter activities between pairs of genes. Another advantage of the algorithm lies in its robustness. Specifically, we observed that even if the accuracy of the absolute values of the estimated half-lives can be influenced by many factors (such as the number of points in the time series, the accuracy of the measurements, the time interval between samples, the choice of the thresholds for the outliers, etc.), the ranking of half-lives is insensitive to the factors mentioned above.

The main disadvantages are the following: DRAGON can work only with time-series under the same experimental condition and cannot handle steady-state values under different conditions. As a general rule a reliable estimate requires at least 10–12 time samples, *i.e.* a number significantly larger than the number of parameters to be estimated (this rule is not obviously always applicable as the required number of points depends strongly on the signal to noise ratio) and a sampling time not larger than the expected average half-life. If no information is available about the correlation of promoter activities, as a rule of thumb, a set of at least 50–100 time series must be processed together in order to have reliable half-lives estimates. One basic hypothesis is that the half-life of a transcript is approximately constant during the time course of the experiment, thus a substantial change of its value would yield an unreliable estimate. These problems can be handled by performing more measurements using a shorter sample time, or by considering moving time windows. The computational overhead can be significant: for a sample of 1000 time series there are 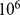 pairs to analyze, requiring a computation of about 150 hours on a medium-speed single-processor machine capable of analyzing 2 pairs per second.

### Conclusion

Our analysis supports and strengthens Shalem *et al.* conclusions about the coordination of transcriptional and mRNA degradation in the cell in response to stress. We have demonstrated that during periodic processes, such as the *P. falciparum* IDC, the reproductive cell cycle and the metabolic cycle, the alternative interplays between changes in mRNA stability and changes in mRNA abundance are activated by periodically switching from a counteracting to a synergistic regulation. In light of these results, the classical vision of periodic processes as the result of serial transcription factor sequential activation, should be re-considered from a broader point of view by including post-transcriptional regulation and coordination.

## Materials and Methods

### Stochastic modeling of expression kinetics and Kalman filtering

We defined as 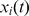 the time profile of the expression of gene 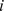 at time 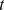. The underlying conservation equation simply stems from the observation that the rate of change of 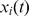 with time, *i.e.* its time derivative 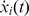, must equal the difference between the production and degradation term. Based on experimental evidence [2], the degradation is well described by a first order term. The dynamics of the 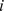-th transcript is therefore described by
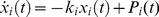
(5)where 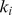 is the mRNA decay rate of 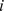-th messenger. This value is linked to the *half-life* 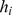 of the transcript by the relation 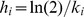. 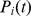 is the 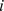-th gene promoter activity regulated by transcription factors. Such regulation occurs by triggering or suppressing the transcription of the 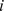-th gene, thus we have 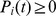. Moreover, the observed measure 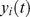 is also a noisy time-series, thus we have
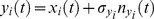
(6)where 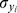 is the standard deviation of measurements white noise 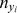 (see supplementary material Text S1 for an example of the identification procedure). We considered a generic pair of expression time profiles characterized by the presence of two terms: a stochastically correlated promoter activity 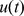 and a gene-specific term 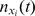. We then considered the case:
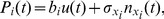
(7)where 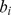 is a scaling factor accounting for the relative contribution to the overall promoter activity regarding gene 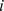. The term 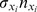 models the part of the promoter activity which is not common to the pair. We model this part by means of a noise term, 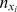 which is assumed to be a white noise. The common part 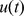 is modeled as a Wiener process:
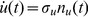
(8)where 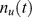 is white noise. Thus 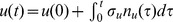. The complete mathematical dynamic model for two transcripts 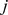 and 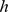, together with their respective measurement equations, is
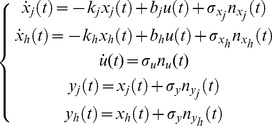
(9)We can rewrite the linear dynamic system (9) using a compact matrix notation
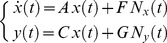
(10)where
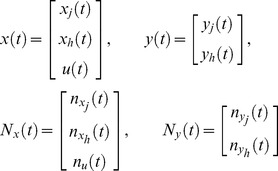
and
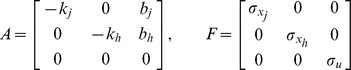


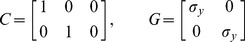
Since the dynamic system (10) is linear, it can be exactly discretized (see [24]) for a given time interval 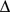, corresponding to the time interval between two consecutive measurements. The 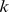-th measurements corresponds to 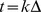, thus in the discretized system we can use 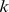 in place of 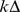, to keep the notation simple.

The solution of the linear dynamic system (10) is
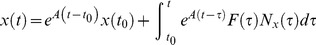
(11)and its discretized form is
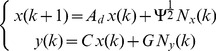
(12)where
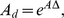
and 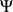 is the covariance matrix defined by
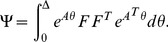
(13)The unknown parameters of the model to be estimated are 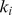, 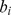, 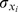, with 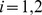, 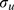 and 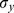. The *state variables* of the system are 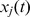, 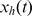 and 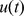. For each given choice of the parameters we used the Kalman filter [25] to estimate of the state variables.

The Kalman filter equation uses a feedback control strategy. It contains a *prediction term* for projecting forward (in time) the current state to obtain the *a priori* estimate, and a correcting term for incorporating a new measurement into the *a priori* estimate to obtain an improved *a posteriori* estimate
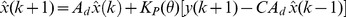
(14)where 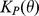 is the prediction Kalman gain that depends on the parameters 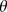 of the stochastic equation.

For each choice of 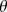 we run the Kalman filter. A probability value is associated to the resulting estimation. These values measures the probability that the current parametrization of the model generates the measured time series. Denoting by 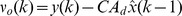 the *innovation* of the stochastic process, 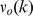 is a sequence of independent gaussian random variables with covariance 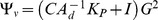. The optimal set 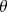 of parameters if therefore chosen according to a *maximum likelihood* criterion as the choice corresponding to the maximum of the *a priori* probability density of the innovation sequence. This corresponds to the minimum of the likelihood function
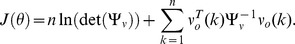
where 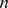 is the number of samples. We are interested in the half life 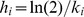 of the 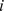-th messenger. To use all the available information and make the method robust with respect to measurement and estimation errors, we have designed the following algorithm (see Supplementary Figure S6):

1. Given a set of 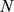 mRNA time profiles, perform the maximum likelihood estimation for *every* pair 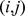 and compute the corresponding 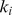 and 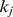.
2. For each pair 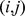 compute the ratio matrix 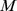 whose elements are the ratios between the half-lives of gene 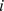 and gene 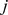. The matrix 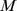 is generally not symmetric due to the presence of outliers and numerical sensitivity. Thus we defined the ratio estimation by row as 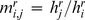 and by column as 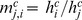. The matrix 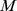 contains all the ratios 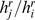 on the 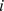-th row, and all the ratios 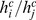 on the 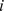-th column. Let us denote 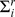 the sum of the 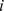
th row, 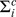 the sum of the 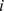 column, and 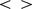 the *mean* operator, that is,
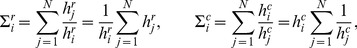
(15)

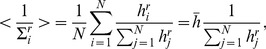
(16)

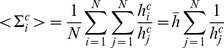
(17)
3. Given 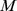, delete outliers to obtain a final matrix 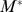. First, compute the probability density (using a smoothing kernel approach) of all the entries 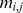 and delete those values below a probability of 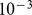 of occurring in the distribution. Second, since ideally 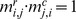, we considered as outliers those pairs such that 
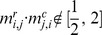
.
4. On the resulting 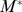 matrix compute for each transcript 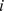 two estimates of its half-life 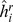 and 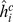, using equations (15), (16)and (17). We obtained
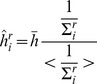
(18)

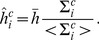
(19)This computation requires the value of 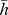. When this value is known for the group of transcripts under analysis the measured value can be used. Otherwise, letting 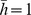 one can obtain half-life values that are relative to the average half-life of the group. However, we have followed a third approach. All the results reported in this paper have been obtained by replacing 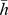 with the geometric mean of 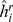 and 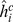, that is
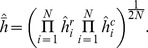
(20)The final estimate of the half-life 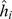 for the 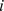-th gene is computed as the weighted average of 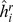 and 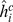 using as weights the respective variances 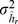 and 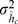 as follows
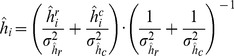
(21)where 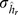 and 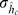 are the standard deviation of the 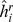 and 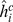, respectively.
5. We considered as a quality index for each estimated half-life 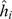 the following:
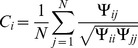
where 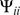, 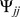 are noise variances of the discrete system (12) and 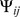 (see equation (13)) is the mutual covariance of the state noise between time series 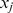 and 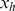. Thus, high values of 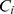 imply the presence of a correlation between 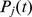 and 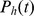 in equation (9). We removed the half-lives having a 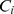 value smaller than the 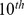 percentile of its distribution.

### Datasets

Public experimental data used throughout the paper are described in Table 1 (experimental half-lives measurements) and in Table 2 (gene expression time series). Pramila *et al.* in [18] and Orlando *et al.* in [19] experimentally measured genome-wide gene expression data during the reproductive cell cycle. We considered the ranking provided by the combined test developed by de Lichtenberg *et al.* [29] for each replicate for the two datasets and, among the list of 1000 genes with highest ranking, we selected those common to all datasets. We ended up with a list of 569 genes that we used for half-life estimation. Tu *et al.* in [20] experimentally measured genome-wide gene expression data during the metabolic cell cycle. We selected 1000 genes with the best periodicity score according to [20]. Of the 1000 genes, DRAGON estimated half-lives are 939.

### Data processing

#### Half-lives determination of genes induced during each stage of *P. falciparum* IDC

Shock *et al.* in [17] experimentally measured genome-wide values of decay rates for each gene in each of the four stages of the IDC. To obtain a single half-life value for each messenger, we performed a k-means clustering of microarray gene expression data [16] by considering 5 stages (according to [16]: early ring, ring, trophozoite, schizont and late schizont). Then, we merged the early ring cluster with the ring cluster to obtain the same stages as in Shock *et al.*. Among the 4488 genes in [16] we chose 1000 genes with the best periodicity score (power signal/power total ratio) according to [16]. Of the 1000 genes, DRAGON estimated half-lives are 967, available experimental half-lives are 675. Both data are available over a set of 616 genes.

### Expression peak timing estimation

To estimate peak timing, for a given noisy gene expression time profile, we preliminary performed the smoothing algorithm presented by Bar-Joseph *et al.* in [30]. The algorithm employs two parameters: grid 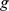 (number of spline curves) and classes 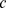 (number of classes to use for clustering). In particular, for Pramila datasets we used 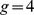 and 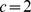, for Orlando datasets we used 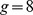 and 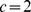, for Tu dataset we used 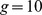 and 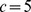, for Malaria dataset we used 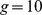 and 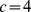.

### Half-life estimations

DRAGON estimated half-lives are provided as supplementary materials Tables S1, S2, S3, S4, S5, S6, S7, described *Supporting Information* section.

Matlab code will be provided upon request.

Additional data and information can be found at web site http://www.dis.uniroma1.it/~farina/dragon.

## Supporting Information

Figure S1 **Functional categories analysis in yeast** ***S. cerevisiae*** **during asynchronous growth measured by three laboratories.** Three genome-wide studies are considered: Grigull *et al.*, Wang *et al.* and Munchel *et al.* (A) Average mRNA half-lives in both studies Wang *et al.* and the Grigull *et al.* datasets for 111 functional categories from the yeast GO Biological Process database (http://www.geneontology.org) that are represented in the set of 2863 transcripts by 5 or more members. (B) compare, in the same way, the Munchel *et al.* and the Wang *et al.* datasets.
(TIF)

Figure S2 **Kinetics of gene induction.** Panels A–B show *in silico* experiments to illustrate some basic features of gene induction kinetics. The reference time profile with unity steady-state is plotted in black. The “ON” and “OFF” regions correspond to the turning “ON” or “OFF” of the promoter activity. (A) Induction kinetic of transcripts having the same half-life value and, as a consequence, the same speed of response. The higher (or lower) steady-state value of the red and blue time profiles is due only to an increased (or decreased) transcription rate. (B) Induction kinetic of transcripts having different half-lives. The time profile plotted in red corresponds to an unstable transcript. It has a faster induction and relaxation profile but a lower steady-state value. By contrast, the blue one has an higher half-life value, resulting in a higher steady state value but a slower response. The example illustrates that, to obtain both a fast response and an high steady-state value, the regulatory strategy must destabilize transcriptionally up-regulated genes.
(TIF)

Figure S3 **Functional categories analysis for DRAGON estimations using** ***P. falciparum*** **IDC data.** Average mRNA half-lives in both studies, DRAGON iestimations *versus* and experimentally measured by Shock *et al.* half-lives, for 12 functional categories from the *P. falciparum* GO annotation database (http://www.geneontology.org) that are represented in the set of 616 transcripts by 5 or more members.
(TIF)

Figure S4 **GO annotations of genes with extreme half-lives in** ***S. cerevisiae*** DNA replication timing requires first the formation of the replication fork, then the production of the needed histones for chromatin assembling: such temporal sequence of events is consistent with a rapid turnover of the replication complex genes and a slow turnover of the histone genes.
(TIF)

Figure S5 **GO annotations of genes with extreme half-lives in** ***S. cerevisiae*** Among unstable genes we also found the G1/S transition cyclins and among stable ones we found G2/M transition cyclins. In this case, the temporal sequence of events is the progression of the cell cycle from DNA replication to mitosis.
(TIF)

Figure S6 **DRAGON algorithm pipeline.**
(TIF)

Table S1 **DRAGON estimated half-lives using alpha30 dataset.**
(XLSX)

Table S2 **DRAGON estimated half-lives using alpha38 dataset.**
(XLSX)

Table S3 **DRAGON estimated half-lives using Orlando replicate 1 dataset.**
(XLSX)

Table S4 **DRAGON estimated half-lives using Orlando replicate 2 dataset.**
(XLSX)

Table S5 **DRAGON estimated half-lives using metabolic cycle dataset.**
(XLSX)

Table S6 **DRAGON estimated half-lives using** ***P. falciparum*** **dataset.**
(XLSX)

Table S7 **DRAGON estimated normalized half-lives using all yeast datasets.**
(XLSX)

Text S1 **Example of parameter estimation through the Kalman filter.**
(PDF)
