# Supplementary figures and images for: Stochastic Modeling of Expression Kinetics Identifies Messenger Half-Lives and Reveals Sequential Waves of Co-ordinated Transcription and Decay
Source: PLoS Comput Biol. 2012 Nov 8;8(11):e1002772. doi: 10.1371/journal.pcbi.1002772 (PMC3493476; doi:10.1371/journal.pcbi.1002772)

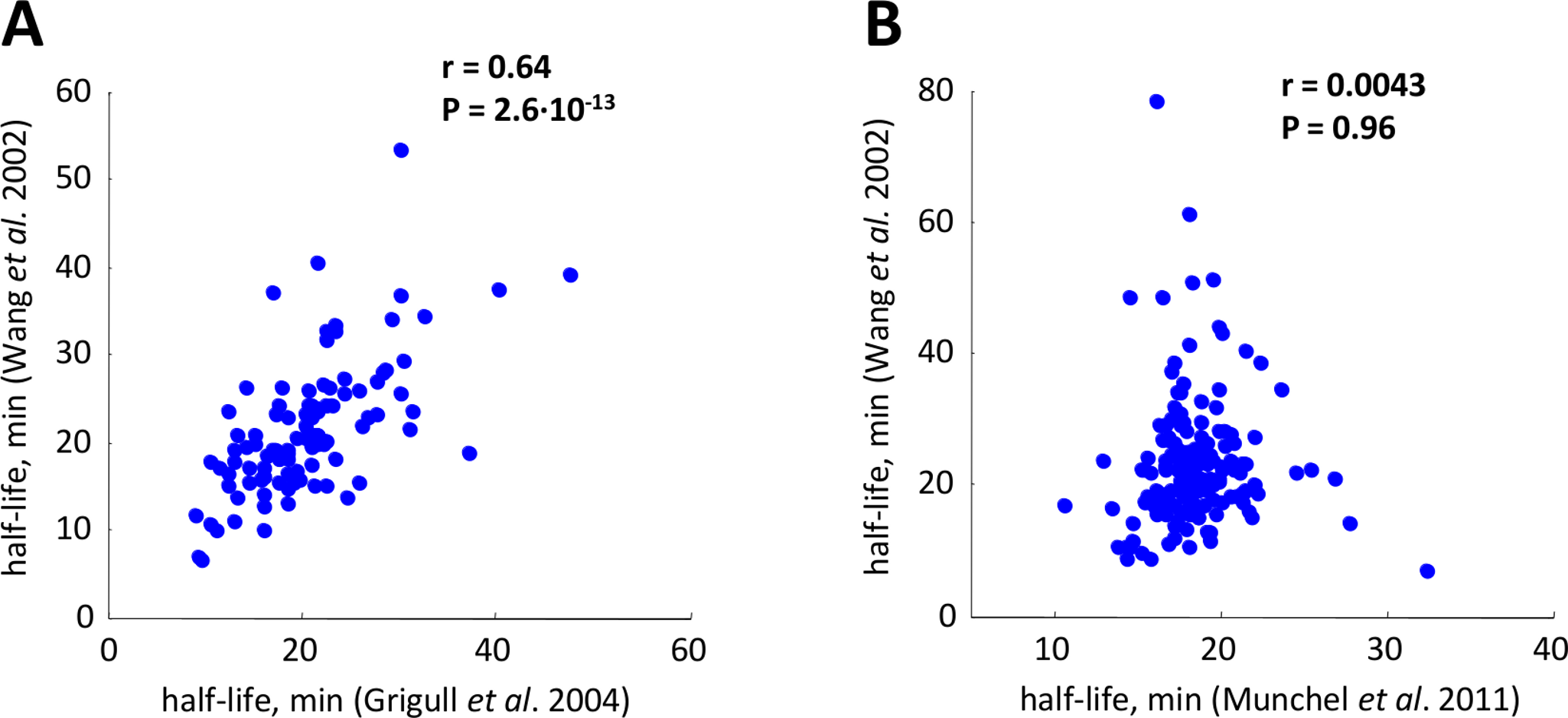

Supplement: Figure S1 — Functional categories analysis in yeast S. cerevisiae during asynchronous growth measured by three laboratories. Three genome-wide studies are considered: Grigull et al., Wang et al. and Munchel et al. (A) Average mRNA half-lives in both studies Wang et al. and the Grigull et al. datasets for 111 functional categories from the yeast GO Biological Process database (http://www.geneontology.org) that are represented in the set of 2863 transcripts by 5 or more members. (B) compare, in the same way, the Munchel et al. and the Wang et al. datasets. (TIF) [file pcbi.1002772.s001.tif]

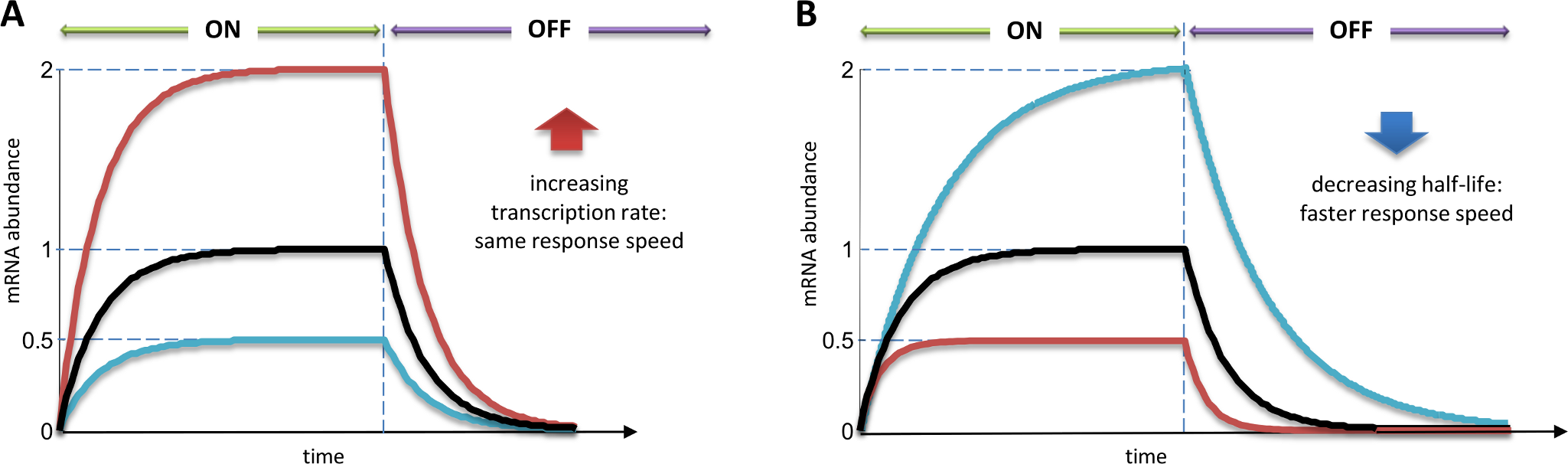

Supplement: Figure S2 — Kinetics of gene induction. Panels A–B show in silico experiments to illustrate some basic features of gene induction kinetics. The reference time profile with unity steady-state is plotted in black. The “ON” and “OFF” regions correspond to the turning “ON” or “OFF” of the promoter activity. (A) Induction kinetic of transcripts having the same half-life value and, as a consequence, the same speed of response. The higher (or lower) steady-state value of the red and blue time profiles is due only to an increased (or decreased) transcription rate. (B) Induction kinetic of transcripts having different half-lives. The time profile plotted in red corresponds to an unstable transcript. It has a faster induction and relaxation profile but a lower steady-state value. By contrast, the blue one has an higher half-life value, resulting in a higher steady state value but a slower response. The example illustrates that, to obtain both a fast response and an high steady-state value, the regulatory strategy must destabilize transcriptionally up-regulated genes. (TIF) [file pcbi.1002772.s002.tif]

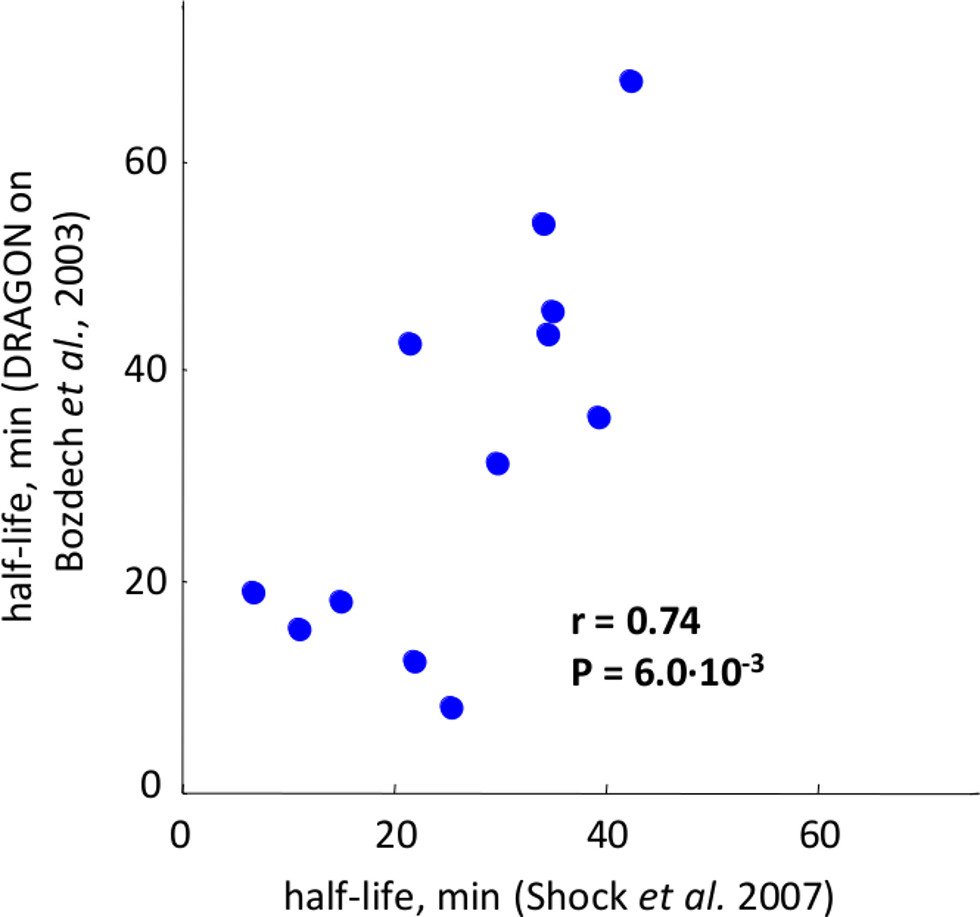

Supplement: Figure S3 — Functional categories analysis for DRAGON estimations using P. falciparum IDC data. Average mRNA half-lives in both studies, DRAGON iestimations versus and experimentally measured by Shock et al. half-lives, for 12 functional categories from the P. falciparum GO annotation database (http://www.geneontology.org) that are represented in the set of 616 transcripts by 5 or more members. (TIF) [file pcbi.1002772.s003.tif]

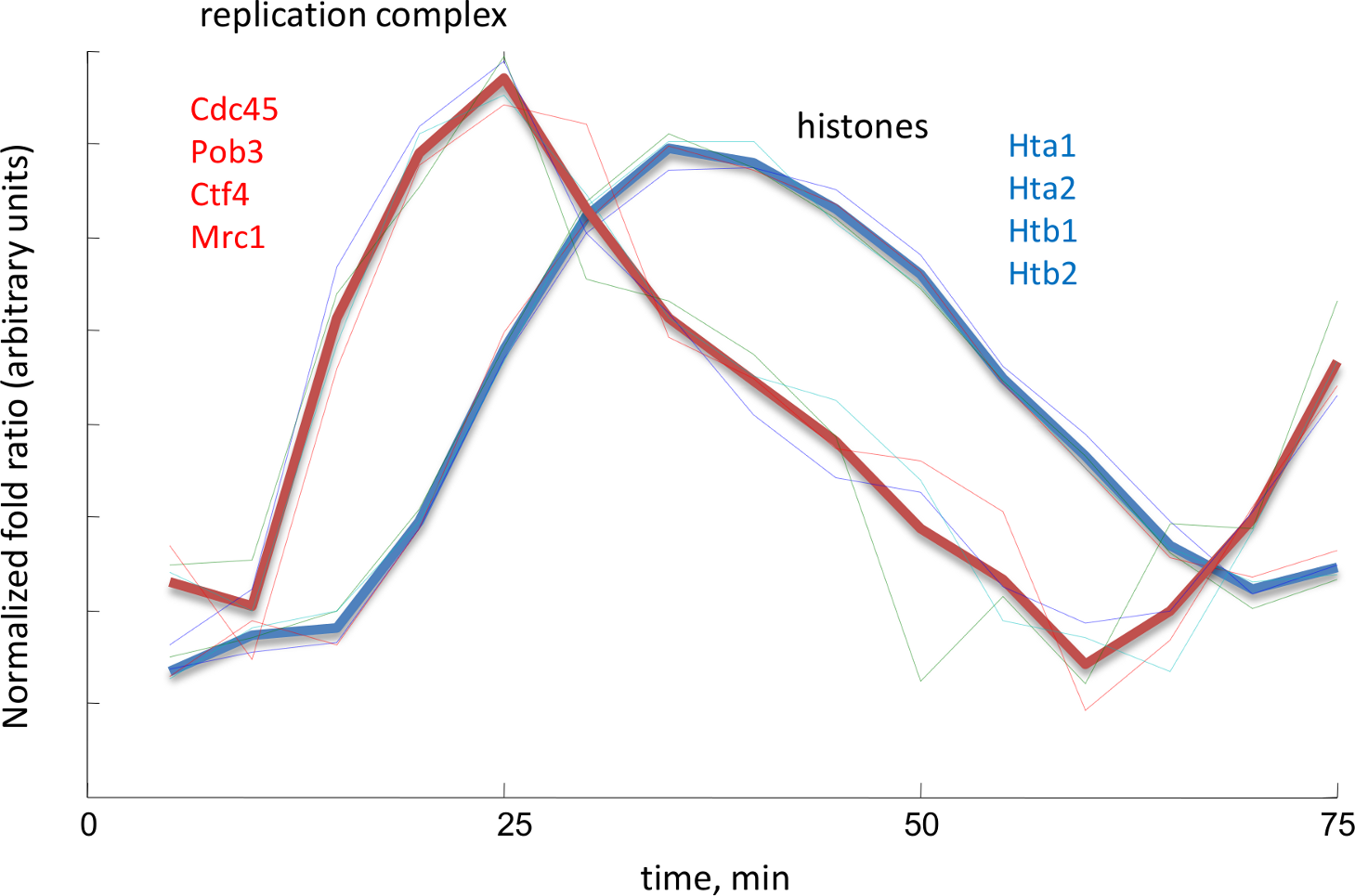

Supplement: Figure S4 — GO annotations of genes with extreme half-lives in S. cerevisiae DNA replication timing requires first the formation of the replication fork, then the production of the needed histones for chromatin assembling: such temporal sequence of events is consistent with a rapid turnover of the replication complex genes and a slow turnover of the histone genes. (TIF) [file pcbi.1002772.s004.tif]

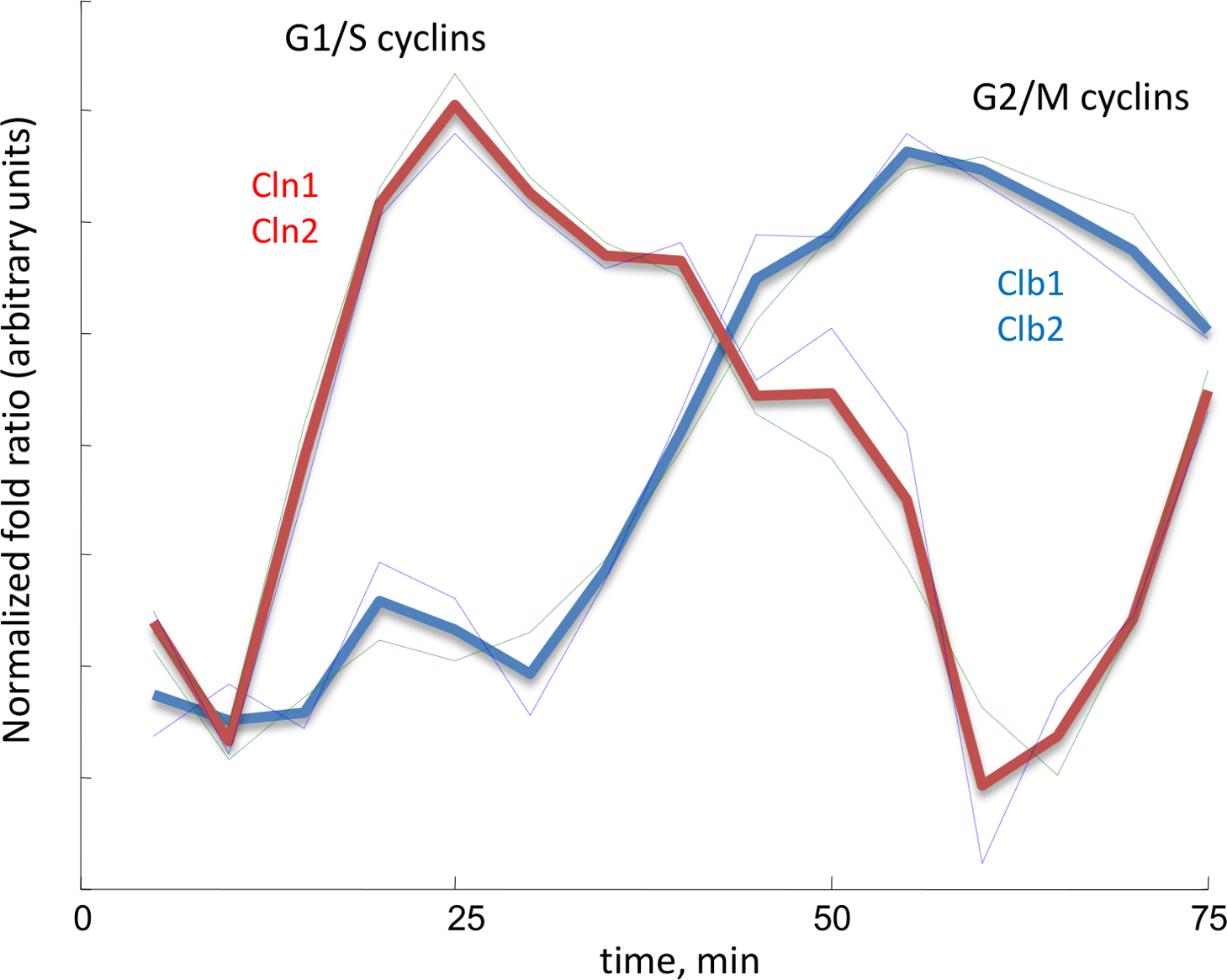

Supplement: Figure S5 — GO annotations of genes with extreme half-lives in S. cerevisiae Among unstable genes we also found the G1/S transition cyclins and among stable ones we found G2/M transition cyclins. In this case, the temporal sequence of events is the progression of the cell cycle from DNA replication to mitosis. (TIF) [file pcbi.1002772.s005.tif]

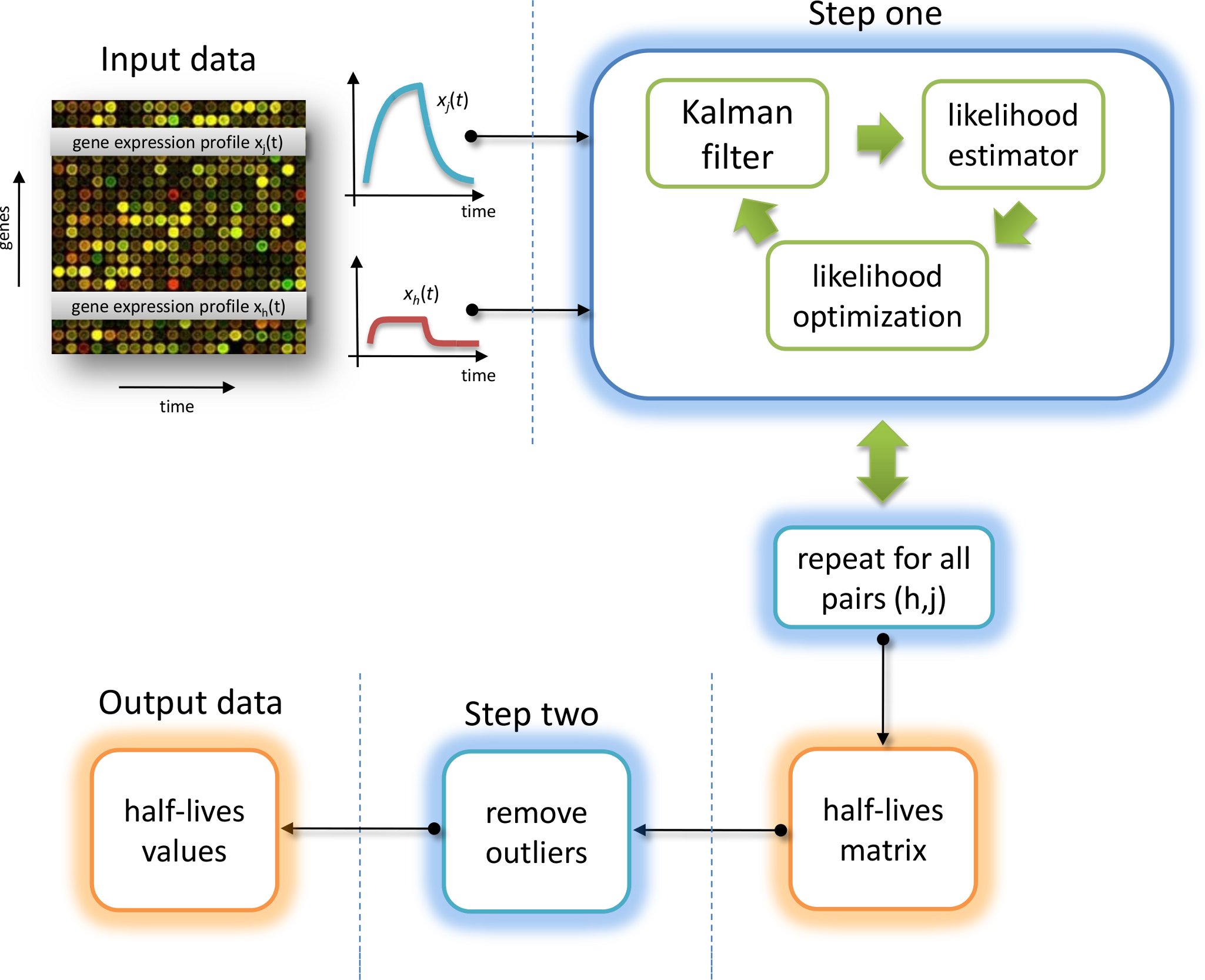

Supplement: Figure S6 — DRAGON algorithm pipeline. (TIF) [file pcbi.1002772.s006.tif]
