# Supplementary material for: Stochastic Modeling of Expression Kinetics Identifies Messenger Half-Lives and Reveals Sequential Waves of Co-ordinated Transcription and Decay
Source: PLoS Comput Biol. 2012 Nov 8;8(11):e1002772. doi: 10.1371/journal.pcbi.1002772 (PMC3493476; doi:10.1371/journal.pcbi.1002772)
Supplement: Text S1 — Example of parameter estimation through the Kalman filter. (PDF) [file pcbi.1002772.s014.pdf]

Supplementary material

August 24, 2012

Example of parameter estimation through the Kalman filter

In this section we aim at giving more insights on the use of the Kalman filter and stochastic modeling to the estimation problem of parameters. To this end, we consider a simplified version of the half-life estimation problem, namely that of estimating the decay rate of a first order system with an unknown input. The system we consider here can be described as

$$\begin{aligned} \dot{x}(t) &= -Kx(t) + u(t) && (1) \\ y(t) &= x(t) + \sigma_y n(t). && (2) \end{aligned}$$

Here, $x(t)$ is the scalar variable that corresponds to the system evolution, whereas $y(t)$ is the available measurement of $x(t)$ and it is affected by a measurement noise $\sigma_y n(t)$. The system input chosen in this example is $u(t) = A\sin(\omega t)$, but it is unknown and thus we assume that it is not possible to use its value to estimate the unknown parameter K. Notice that the measurement equation contains a stochastic term. We suppose to know σ_y and the output sequence $y(t)$ at discrete time points. In figure the time courses of $x(t)$ and $y(t)$ are plotted for the following choice of parameters: $K = 0.0087$, that corresponds to a half-life $h = \log(2)/K = 80$, $\omega = 0.2$, $A = 0.05$, $\sigma_y = 0.10$, and sampling time $\Delta = 1$.

Since we require that the Kalman filter cannot use the true $u(t)$, the input can be modeled as stochastic process. In particular, every possible trajectory of $u(t)$ is modeled as a realization of a Wiener process, thus we can rewrite system (1)-(2) in the appropriate stochastic formalism as

$$\begin{aligned} dx_t &= -Kx_t dt + \sigma_x dW^1 && (3) \\ dy_t &= x_t dt + \sigma_y dW^2, && (4) \end{aligned}$$

where dW^1 and dW^2 are two independent Wiener processes. In other words, the equations used to generate the time series to analyze are (1)-(2), whereas the equations used to build the filter are (3)-(4).

Since we assume that measurements are taken at discrete intervals, we integrate (3)-(4) in the interval Δ. The resulting discrete-time system is

$$\begin{aligned} x(k+1) &= -A_K x(k) + FN_x^k && (5) \\ y(k) &= x(k) + GN_y^k, && (6) \end{aligned}$$

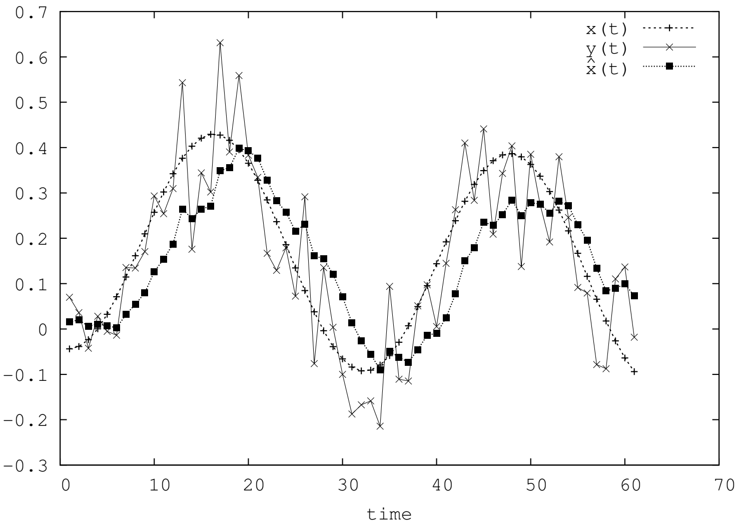

Figure 1: A sample evolution of the system: comparison of real value $x(t)$, measured value $y(t)$, and the value $\hat{x}(t)$ estimated by the Kalman filter, all sampled every unit of time. Here $K = 0.0087$, $\hat{K} = 0.0080$.

where N_x^k and N_y^k are independent random variables with normal distribution. Straightforward derivations yield $A_K = e^{-K\Delta}$ and, for the amplitudes of the noises F and G,

$$F = \Psi^{\frac{1}{2}} \tag{7}$$

$$\Psi = \sigma_x^2 \int_0^{\Delta} e^{-2K\theta} d\theta = \frac{\sigma_x^2}{2K} \left(1 - e^{-2K\Delta}\right) \tag{8}$$

$$G = \sigma_y. \tag{9}$$

Notice that in order to have all the parameters we need σ_x, the amplitude of the input random process that we use in the place of $u(t)$. Even if in general σ_x may be an unknown parameter to estimate in the same way as K, to keep the example simple we suppose instead that σ_x is known, and we compute it as the average energy of the input $u(t)$

$$\sigma_x^2 = \frac{1}{2\pi} \int_0^{2\pi} \left(A \sin(\omega t)\right)^2 dt. \tag{10}$$

With this choice $\sigma_x dW^1$ will have the same average quadratic amplitude as $u(t)$, and this is equivalent to saying that we do not know $u(t)$ but only its average energy. With the values used here, we have $\sigma_x = 0.0258$ (of course the method should work even if an approximate estimate of σ_x is used instead of the true value).

For each choice of the unknown parameter K we are equipped with all the parameters A_K, F and G occurring in equations (5)-(6), thus we can write the

equation of the standard Kalman filter for the variable $\hat{x}(k)$ which is the filter estimate for $x(k)$, as

$$\hat{x}(k+1) = A_K \hat{x}(k) + \bar{K}_\infty \left(y(k+1) - A_K \hat{x}(k) \right). \tag{11}$$

The first term in the right-hand side is a projection of $\hat{x}(k)$ one step farther, whereas the second term is a correction based on the comparison between the measured value $y(k+1)$ and the predicted value $A_K \hat{x}(k)$. This correction is multiplied by the *Kalman gain* $\bar{K}_\infty$, which is computed only once solving the equations

$$\bar{K}_\infty = P_\infty / G^2 \tag{12}$$

$$P_\infty \left(1 + (A_K^2 P_\infty + \Psi)/G^2 \right) = A_K^2 P_\infty + \Psi, \tag{13}$$

where P_∞ is the steady-state covariance of the estimation error $e(k) = x(k) - \hat{x}(k)$, that is, $P_\infty = \lim_{k \to \infty} E(e^2(k))$, where $E()$ denotes the expected value. Solving (12) we obtain $\bar{K}_\infty$ and we can implement the Kalman filter (11) for each choice of the unknown parameter K. We still need a likelihood functional whose maximum corresponds to the value of K that is most likely given the measurement sequence $y(k)$. To this end, we consider the *innovation process* $\nu(k)$, defined as

$$\nu(k+1) = y(k+1) - A_k \hat{x}(k), \tag{14}$$

which is the difference between the real and predicted measurement. Based on results of the theory of stochastic processes, we have that $\nu(k)$ is a random variable with normal distribution. The steady state variance of the corresponding stochastic process $\Psi_\infty^\nu = \lim_{k \to \infty} E(\nu^2(k))$ can be computed and it is

$$\Psi_\infty^\nu = P_\infty + G^2. \tag{15}$$

Notice that for each choice of K we can now compute the sequences $\hat{x}(k)$ and $\nu(k)$, for $k = 1, \ldots, N$, where N is the umber of sampled measurements. The likelihood functional that we use to find K is the probability that the sequence $\nu(k)$ obtained for a given choice of K is indeed a random variable with the predicted variance Ψ_∞^ν. That is we use the likelihood functional $V(K)$ defined as

$$V(K) = \prod_{i=1}^{N} \frac{1}{\sqrt{2\pi \Psi_\infty^\nu}} e^{-\frac{\nu^2(k)}{2\Psi_\infty^\nu}}. \tag{16}$$

An optimization algorithm is used to find the maximum of $V(K)$ over a range of K. For the numerical simulations of this example we have chosen $K \in [0.0069,\ 0.6931]$, corresponding to a range of half-lives [1, 100]. Figure illustrates the predicted $\hat{x}(k)$ of the Kalman filter for one trace generated from the equation (1)-(2). In this case, the optimization found the value $\hat{K} = 0.0080$, corresponding to a half-life 86.9, while the true value was $K = 0.0087$ (half-life 80.0).

Robustness of the DRAGON estimates

The algorithm uses two thresholds (step 3 in the Materials and Methods section) for the selection of the elements m_{ij} in the ratio matrix M. It is important to

verify their impact on the resulting estimates. We briefly comment on this, comparing the Pearson correlation between our estimation of half-lives and the measurements of Shock *et al.* for *P. falciparum* when these thresholds are changed. The first threshold is a probability value used to delete the elements m_{ij} in the tails of the distribution. Actually, this is a safeguard against values completely out of range due to numerical artifacts generated by the optimization algorithm. We found no significant difference in the Pearson correlation when this threshold was 0 (no m_{ij} deleted). The second threshold is on the range of the product $m_{ij} \cdot m_{ji}$. A value close to 1 indicates that the same maximum has been found for the likelihood functional when swapping the time series. A product far from 1 indicates that two distinct maxima have been found, and the information related to that pair of time series is probably not useful and it should be discarded. The threshold is denoted as k, and the admissible interval must be in the range $[1/k, k]$. In the case of the *P. falciparum* dataset, with $k = \infty$ (no selection of matrix elements), we get a Pearson correlation 0.41 (P-value 10^{-25}) instead of 0.6 (P-value 10^{-61}) obtained with $k = 1$. Larger values of k have lesser effect: with $k = 2$ the correlation drops only to 0.53 (P-value 10^{-43}). We can conclude that deleting non symmetric entries of M is indeed important but the precise tuning of the parameter is not crucial in order to get reliable estimates.
